# Alternative Splicing of Alpha- and Beta-Synuclein Genes Plays Differential Roles in Synucleinopathies

**DOI:** 10.3390/genes9020063

**Published:** 2018-01-25

**Authors:** Ana Gámez-Valero, Katrin Beyer

**Affiliations:** Department of Pathology, Germans Trias i Pujol Research Institute, Badalona, 08916 Barcelona, Spain; avalero@igtp.cat

**Keywords:** α-synuclein, *SNCA*, β-synuclein, *SNCB*, alternative splicing, functional splice variants, differential expression

## Abstract

The synuclein family is composed of three members, two of which, α- and β-synuclein, play a major role in the development of synucleinopathies, including Parkinson’s disease (PD) as most important movement disorder, dementia with Lewy bodies (DLB) as the second most frequent cause of dementia after Alzheimer’s disease and multiple system atrophy. Whereas abnormal oligomerization and fibrillation of α-synuclein are now well recognized as initial steps in the development of synucleinopathies, β-synuclein is thought to be a natural α-synuclein anti-aggregant. α-synuclein is encoded by the *SNCA* gene, and β-synuclein by *SNCB*. Both genes are homologous and undergo complex splicing events. On one hand, in-frame splicing of coding exons gives rise to at least three shorter transcripts, and the functional properties of the corresponding protein isoforms are different. Another type of alternative splicing is the alternative inclusion of at least four initial exons in the case of *SNCA*, and two in the case of *SNCB*. Finally, different lengths of 3’ untranslated regions have been also reported for both genes. *SNCB* only expresses in the brain, but some of the numerous *SNCA* transcripts are also brain-specific. With the present article, we aim to provide a systematic review of disease related changes in the differential expression of the various *SNCA* and *SNCB* transcript variants in brain, blood, and non-neuronal tissue of synucleinopathies, but especially PD and DLB as major neurodegenerative disorders.

## 1. Introduction—the Synuclein Family

The three members of the synuclein family, α-, β-, and γ-synucleins (AS, BS, and GS) are presynaptic proteins, encoded by highly homologous genes, which have been found, so far, only in vertebrates [1]. Whereas AS and BS show a homology of 78%, GS shares 60% of the AS sequence [2,3]. Synucleins are small, intrinsically disordered proteins without a stably folded structure under physiological conditions, and are expressed primarily in neural tissue and in certain tumors [4,5]. A typical structural feature of synucleins is the presence of a repetitive amino acid motif throughout the N-terminal, and acidic stretches within the C-terminal region of the protein [6]. Their characteristics as disordered proteins convert synucleins into multifunctional proteins [7]. Whereas AS and GS easily form fibrils under various but similar conditions [8], BS fibrillates with much more difficulty [9]. Although AS is known as a key factor in the development of synucleinopathies [10], the presence of AS, BS, and GS has been detected in vesicular-like lesions at presynaptic axon terminals in the hippocampal dentate and CA2/3 regions, and in hilar neurons [11]. In the synapses, synucleins may compensate each other in their functions. This compensation process was determined in an AS knockout mouse model, where synaptic function was not compromised with aging [11]. Nevertheless, in triple knockout mice, shutting down all three synucleins, a gradual decline of synaptic function was observed, and its manifestations were detectable with aging [11,12,13]. However, the three synucleins show differential involvement in synaptic function. An important, but not significant decrease of striatal dopamine levels has been observed in aging AS null mice, and in AS/BS double knockout mice [14,15]. Instead, neither AS/GS nor GS null mice showed any synaptic alterations, indicating that AS plays the most important role in synaptic function, and that BS is able to rescue this function in its absence [16]. Finally, a recent study revealed that each synuclein has a distinct set of functions, which do not completely overlap. Only some functions can be compensated by the other synucleins [17]. For instance, AS is responsible for the maintenance of dopamine levels in the nigrostriatal system, and BS for motor functions within other neuronal systems [17]. However, the fact that the synuclein-null mice described above are viable suggests that synucleins are not essential components of the neurotransmitter release machinery, but are rather involved in the long-term regulation and maintenance of nerve terminal function [15,16,17,18,19]. Since alternative splice variants in the context of neurodegenerative disease have not been reported for the GS gene, it will not be included in this review. 

During the last decades, the synuclein family has aroused increasing attention, because AS especially is primarily involved in the pathogenesis of synucleinopathies [20]. These include Parkinson’s disease (PD) and dementia with Lewy bodies (DLB) characterized by the presence of intraneuronal Lewy bodies (LB) and belonging together to the group of LB diseases, and multiple system atrophy (MSA), where α-synuclein accumulates in glial cytoplasmic inclusions (GCI). 

Parkinson’s disease is the most common progressive movement disorder in the elderly, and DLB is the second most frequent cause of dementia after Alzheimer’s disease (AD) [21]. Whereas the substantia nigra is the most affected brain area in PD [22], widespread distribution of LBs throughout almost all brain areas is found in DLB [23]. Post-mortem neuropathological studies have shown that LB pathology can be present alone in its pure form (pDLB), or by the mixture of LB and concomitant AD pathology which constitutes common variant of DLB (cDLB) [24,25,26]. About 20–50% of PD patients develop dementia (PDD) after no less than 10 to 15 years following a PD diagnosis [27,28]. The morphological substrate, such as AS immunoreactive LBs and Lewy neurites, is the same in both PDD and DLB. However, some neuropathological differences between DLB and PDD, including a higher striatal A-β load, independently from cortical AD pathology in DLB rather than in PDD, have been suggested [29,30,31]. Despite the fact that the clinical course between DLB and PDD differs, it is difficult to detect neuropathological differences between the two conditions. In DLB, the first clinical symptoms are linked to dementia with motor signs appearing concomitantly, or less than one year after onset of cognitive symptoms. In addition, the disease often progresses rapidly with duration of between 6–10 years [25,32]. On the contrary, PD patients can develop dementia late, and survive up to 30 years [33]. 

### 1.1. α-Synuclein—Structure and Function

**Figure 1:**
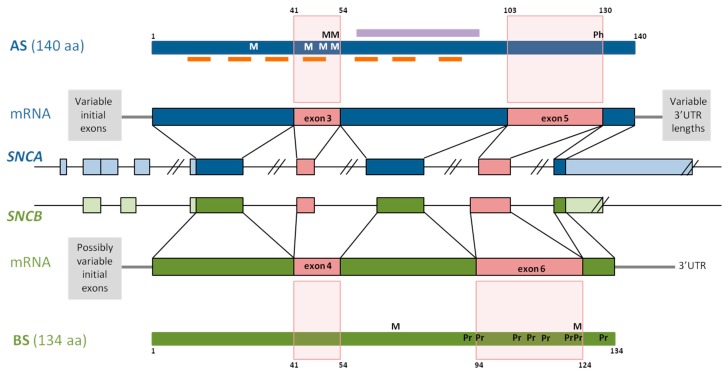
Schematic representation of the *SNCA* (blue) and *SNCB* (green) genes. Coding regions are colored in dark and untranslated regions in light. Exons affected by alternative splicing are colored in pink. The corresponding transcripts are shown above or below their respective genes, as are the resulting proteins. Light pink squares mark the protein regions that are deleted as a result of in-frame splicing. Short orange bars represent the seven imperfect repeats, the violet bar represents the non-amyloid component (NAC) domain. M, shows the location of the different mutations in both α-synuclein (AS) and β-synuclein (BS); Ph, is the main phosphorylation site at S129 in AS; Pr, proline residues along the C-terminal of BS.

The AS gene *SNCA* is located on chromosome 4q21.3-q22, contains five coding exons, and at least three additional 5’ exons alternatively included in the different *SNCA* transcripts (Figure 1). The main transcript gives rise to the aggregation prone 140 amino acid protein AS [34]. The primary structure of AS comprises residues 1–60 as N-terminal region, residues 61–95 as central region, and residues 96–140 as C-terminus [35]. Seven imperfect repeats of 11 amino acids with a highly conserved hexameric motif (KTKEGV) span the N-terminus and central region (Figure 1), forming an N-terminal helix that binds AS to membranes [2,36,37,38], and a central helix responsible for protein–protein interactions. The central region, first identified as the non-amyloid component (NAC), is the most aggregation-prone part of AS (Figure 1) [39,40], and the 12-amino acid stretch between residues 71 and 82 is necessary and sufficient for AS fibrillation [41]. Finally, the C-terminus is highly enriched in acidic residues [42], conferring a negative charge to this protein part at physiological pH. The shortening of the C-terminal, accompanied by the deletion of negatively charged amino acid residues, results in an increase of its net charge, and consequently, in an elevated aggregation propensity [43]. 

Since AS is a multifunctional protein, it participates in different cellular processes [7]. Through interaction with membranes, AS is involved in membrane channel formation and modification of their transport activity [44], in synaptic vesicle release and trafficking [45,46], and in positive and negative regulation of neurotransmitter release [47]. α-Synuclein is also implicated in the activation of microglia [48], it associates with mitochondria under stress conditions [49], and regulates the neuronal apoptotic response, protecting neurons from several apoptotic stimuli [50]. α-Synuclein also interacts with numerous proteins and other binding partners [51,52,53], including synaptosomal proteins [54], mitochondria associated membranes [55], molecular chaperones [56,57], and synapsin III [58]. 

Despite all these diverse functions, it is the increased abnormal oligomerization and aggregation of AS which is considered the key event preceding LB formation in the pathogenesis of synucleinopathies [10,20,59]. Intraneuronal LBs are found in vulnerable areas [22,60] and the accumulation of small presynaptic AS aggregates correlates with dendritic spine loss and associates with early neurodegeneration in DLB [61]. Lewy body-related pathology initiates in defined brain regions, with disease progression it spreads gradually throughout the whole brain [62,63,64,65]. 

Shortly after the description of AS as main LB component in 1997 [66], three missense mutations, A30P and A53T in familial PD [67,68], and E46K in DLB [69], were identified in the *SNCA* gene. Later, duplication and triplication of the *SNCA* locus were suggested to be responsible for elevated AS levels in the brain, promoting oligomerization and aggregation of the protein [70,71,72]. Finally, only a few years ago, three additional mutations have been identified: H50Q in PD with a family history of parkinsonism and dementia [73,74], G51D causing a form of PD with unusual clinical, neuropathological, and biochemical features [75,76], and A53E as the first AS mutation in MSA [77]. A lot of effort has been made to identify the effect of *SNCA* mutations on the protein and its functionality, and multiple studies have shown that *SNCA* mutations do not alter the structure of AS fibrils, but affects their relative stability and conformation [78].

### 1.2. β-Synuclein—Structure and Function

The BS gene, *SNCB*, is located on chromosome 5q35.2, and contains similarly to *SNCA*, five coding exons and at least two additional 5’exons (Figure 1). BS is a 134 amino acid protein, and its N-terminal shows a 90%-homology with the AS N-terminal. Together with AS, BS expresses at synaptic terminals, and has not been found neither in LBs nor in Lewy neurites [79]. Despite structural similarities, BS has very different self-association and aggregation properties when compared to AS [8,80,81,82]. Indeed, it has been shown that BS is able to inhibit AS aggregation [8,82] and to reduce AS toxicity. These opposing characteristics are due to the amino acid sequence and structural differences of BS comprising both the central region and the C-terminal. The central region of BS is characterized by an 11 amino acid deletion, the larger number of proline residues and their particularly distinctive distribution in comparison with AS (Figure 1) [83]. Analysis of its structural characteristics revealed that BS has an increased α-helical propensity that, in addition to the lack of the central hydrophobic cluster, may stabilize the intrinsically disordered state of BS [83]. Thus, low ratios of β-structured intermediates are responsible for the inhibition of oligomerization described for BS. Moreover, due to the elevated proline content within the C-terminal of BS (Figure 1), transient polyproline II conformations have been described at this region. Polyproline II helices are involved in transcription, cell motility, self-assembly, and elasticity, and this conformation is favorable for protein–protein and protein–nucleic acid interactions [84]. The extended conformation and flexibility of the polyproline II structure not only resembles the proline-rich sequences of synaptic vesicle related proteins [83], but is also responsible for the anti-amyloidogenic characteristics reported for BS [85]. 

In addition to anti-aggregation and anti-amyloidogenic properties, levels of post-transcriptional modifications are also different in AS and BS. Specifically, isoaspartate formation, a post-translational modification due to the lability of asparagines and aspartic acid residues, accumulates with aging. Isoaspartate content is notably lower in BS when compared to AS, and BS may prevent isoaspartate accumulation in AS [86]. 

β-Synuclein inhibits α-Synuclein fibril formation, aggregation, and neurotoxicity in a dose-dependent manner [87,88], and through direct interaction with AS [8,87,89,90,91,92]. As a result, BS is incorporated into the transient oligomeric intermediates of AS, stabilizes them, and prevents their conversion into stable fibrils [8]. In vitro, specific peptides within the N-terminal of BS are responsible for the inhibition of fibril formation [92,93], while a specific 10 amino acid peptide derived from the central region of BS inhibits AS oligomer formation [93].

However, recent studies indicate that BS may also undergo toxic gain-of-function, as they have shown that BS may induce neurotoxicity in primary neurons, but also dopaminergic neurons [94,95]. In this context, BS was found to induce mitochondrial pathology, to form membrane channels and to accumulate in small cytosolic proteinase K resistant inclusions [94,95]. Moreover, slight changes in pH promote BS fibril formation, due to the acidic residues situated along the protein [96].

So far, two BS mutations, V70M and P123H, have been identified in sporadic and familial DLB, respectively [97]. Whereas both are involved in lysosomal pathology, P123H abolishes a proline residue, inducing significant changes within the polyproline II structure by compaction of the C-terminus [98]. This change is sufficient to abolish the non-amyloidogenic characteristics of BS, to convert it into a neurotoxic species, and to induce the formation of neuritic pathology [84,97,99]. 

## 2. *SNCA* Alternative Splicing and Its Role in Synucleinopathies

Alternative splicing consists of the inclusion of different exons in the mature mRNA molecule. The result is the generation of various transcripts from one single gene [100]. More than 90% of human genes undergo alternative splicing [101], having the most important impact on protein diversity and explaining the discrepancy between the number of protein-coding genes, estimated at 24000, and the number of proteins that are thought to be synthesized, exceeding that number about 4 times [100,102,103,104]. Thus, alternative splicing greatly enhances transcriptomic and proteomic diversity, and is, at the same time, the major source for the phenotypic complexity in higher eukaryotes [100,104].

The four main forms of alternative splicing are: (i) exon skipping, where one or more exons can be spliced out of the mRNA; (ii) alternative inclusion of starting exons at the 5’ untranslated region (UTR), (iii) alternative selection of 3’UTR; and (iv) intron retention, in which an intron can remain in the transcript. Although there are other, less frequent and complex events resulting in alternative transcripts of a gene, three of the above listed processes have been observed for the *SNCA* gene. 

The National Center for Biotechnology Information (NCBI) provides information related to genetic variability and expression. To face the continuous input of new data, NCBI has engaged in the Locus Reference Genomic (LRG) project, creating genomic sequences to be used as reference standards for establishing conventions for numbering exons and introns, and for defining the coordinates of other variations.

Temporarily, before creating the current version of the *SNCA* refseqgene (NCBI accession: NG_011851.1), GenBank data indicated that *SNCA* contains more than six exons (NCBI accession: NG_011851; now removed), as also demonstrated by numerous *SNCA* transcripts including different initial exons (Figure 1). However, according to the refseqgene sequence of *SNCA* provided by NCBI, *SNCA* is a six exon gene, contains five coding exons, with the coding sequence beginning in exon 2. 

In regard to *SNCA*, the existence of at least four different 5’UTR exons has been confirmed, and exons 3 and 5 are also alternatively spliced. A set of at least four transcripts for each of the four 5’UTR differing isoforms, consisting of an exon 2–6 containing transcript, an exon 5 lacking transcript, an exon 3 lacking transcript, and an exon 3 and exon 5 lacking transcript, have been reported [105]. Additionally, *SNCA* transcripts with at least five different 3’UTR have been also identified [106]. 

### 2.1. 5’ Untranslated Region Splicing

**Figure 2:**
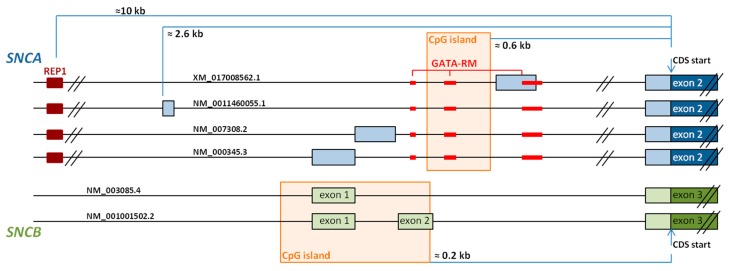
Schematic representation of the 5’UTR of *SNCA* (blue) and *SNCB* (green). *SNCA* contains multiple 5’UTR exons, the transcription of four mRNAs differing in their initial exons has been confirmed and the remaining are predicted to exist [107]. Three regions within *SNCA* intron 1 contain GATA recognition motifs (GATA-RM; red squares) [108]. A CpG island spanning 600 bp, also located in intron 1, is shown in an orange square (calculations carried out at [109]). *SNCB* 5’UTR contains less exons than *SNCA* [110]. The transcription of two of the six *SNCB* transcripts with different initial exons has been confirmed, one of them including only exon 1 and the other exons 1 and 2. A CpG island spanning 900 bp includes a region upstream to the *SNCB* transcription start. It is shown in an orange square (estimations performed at [109]).

The 5’UTR of the *SNCA* gene contains more than 10 different initial exons to be included alternatively into *SNCA* mRNA [107]. Of these, we have analyzed the expression of four different transcripts, NM_0011460055.1, NM_000345.3 (the main *SNCA* transcript), NM_007308.2, and XM_017008562.1, in brain and blood. Whereas NM_000345.3 and NM_007308.2 are the mostly expressed transcripts, XM_017008562.1 is only found in brain (unpublished data, Figure 2).

Comprehensive analysis of the human genome has shown that an elevated number of genes expresses alternative 5’UTR by using multiple promoters [111]. When regulatory motifs are included in specific transcripts and not in others, 5’UTRs may determine tissue specific expression of a transcript [111]. Although, so far, there are no studies available that correlate *SNCA* 5’UTR-specifc transcripts with promoter activity, some data indicate that *SNCA* is regulated by GATA transcription factors and a CpG island, as well as by a complex microsatellite repeat at about −10 kB upstream of the *SNCA* transcription start. 

First, Scherzer and colleagues [112] showed that GATA-1 activates *SNCA* transcription in erythroid precursor cells, and that GATA-2 may replace GATA-1 in absence of the latter during erythropoiesis [108]. In neurons, including dopaminergic neurons in the substantia nigra, GATA-2 can similarly substitute GATA-1. GATA transcription factors predominantly bind to motifs located in *SNCA* intron 1 (Figure 2), and are responsible for the trans-activation of *SNCA* transcription [112].

A CpG island, also located in intron 1, was described later (Figure 2) [113], and its methylation levels differ significantly between the different brain areas [114]. Various studies have shown that *SNCA* promoter methylation is decreased in PD [113,115,116] and also in DLB [117], and that methylation levels increase in a dose-dependent manner after treatment with l-dopa [116]. 

A third element to be involved in the regulation of *SNCA* transcription is REP1, located about 10 kb upstream of the *SNCA* transcription start site [118,119]. REP1, a complex polymorphic microsatellite repeat, is essentially triallelic, and compared with the intermediate-length allele, the longest allele is associated with high *SNCA* expression levels, whereas the shortest allele with low *SNCA* expression [120]. Correspondingly, long REP1-alleles are associated with increased risk of developing late-onset idiopathic PD [121,122].

Our knowledge is constantly increasing, and multiple *SNCA* transcripts differing in their 5’UTR have been identified, so that over the following years, the role of the different regulatory elements on the expression of the various transcripts remains to be determined. 

### 2.2. Exon Skipping

Exon skipping is the most frequent type of alternative splicing, and constitutes up to 40% of all splicing events. The exclusion of an exon may result in in-frame splicing, characterized by the deletion of one or more exons retaining the original reading frame, producing shorter but functional proteins. This type of splicing permits the prediction of possible structural and functional changes. On the other hand, the exclusion of an exon may alter the open reading frame, leading to proteins that contain premature termination codons. These proteins are recognized and targeted for degradation by nonsense-mediated decay [123,124].

Four different AS proteins arise from alternative in-frame splicing of exon 3, exon 5, or both: AS140, AS112, AS126, and AS98, where the number indicates the amino acid content of each isoform [125,126]. Whereas AS140 is the whole protein, AS112 lacks the sequence corresponding to exon 5, AS126 lacks exon 3, and in AS98, neither of them is included. Figure 1 illustrates how the splicing-out of these protein parts impacts the overall structure of the protein, giving rise to specific structural differences among these proteins, permitting accurate prediction of their functions. 

#### 2.2.1. In-Frame Splicing of Exon 5

Exon 5 lacking *SNCA* transcripts give rise to AS112. This isoform lacks amino acids 103–130 at its C-terminal (Figure 1), shortening the least organized part of the protein [43,127,128]. The AS C-terminal lacks a defined secondary structure [129,130], and truncation of this protein part bears protein variants with even higher aggregation propensity than full length AS, which are able to seed the AS aggregation [131]. Moreover, three glutamic acid and one aspartic acid residue within the sequence, corresponding to exon 5 of the AS C-terminal region, are responsible for the reduced aggregation propensity of this protein part [131]. Accordingly, Levitan and colleagues [43] showed later that the kinetics of AS aggregation depends on the charge of its C-terminal, with high content of negative amino acid residues lowering the aggregation rate of AS [43]. With the deletion of exon 5, the amount of negative amino acid residues diminishes increasing AS net charge from −9 to 1, a characteristic that, at the same time, could increase the aggregation propensity of this AS isoform [105]. 

Another important structural implication of splicing out exon 5 is the loss of the amino acid S129, that represents the major AS phosphorylation site. Phosphorylation at S129 plays a primary role in the development of Lewy pathology, since phosphorylation at S129 has been involved in the regulation of AS clearance, aggregation, and toxicity (reviewed in [132]). Whereas in normal brain, only about 5% of AS is phosphorylated at S129, almost 90% of AS found in early aggregates and LBs is phosphorylated at that amino acid [133,134,135]. Therefore, the lack of S129 could be decisive for the aggregation properties of AS112. Correspondingly, aggregation experiments for the different AS isoforms in HEK239T cells revealed that transfection with AS112 only, did not enhance the formation of aggresomes or multiple aggregates [136]. The aggregation of AS was not enhanced when HEK239T cells were co-transfected with either 80% of AS140 or 20% of AS112 [136]. By contrast, another study carried out in a yeast model showed that, although expression of AS112 alone displayed marginal toxicity, the co-expression of both AS140 and AS112 enhanced the toxicity of AS140 [137]. 

A study, carried out by Kalivendi and colleagues [138] revealed that alternative splicing of the *SNCA* gene is enhanced by parkinsonism, inducing toxins. Mice treated with 1-methyl-4-phenyl-1,2,3,6-tetrahydropyridine (MPTP) express AS112, which is responsible for proteasomal dysfunction, especially in the substantia nigra of those mice. A similar effect of AS112 was observed in a human dopaminergic cell line, where cell death occurred after proteasome blocking [138]. In an extension of their study, the same authors reported that AS112 presents temperature-dependent aggregation propensity [139], but loses chaperone activity inherent to AS140 [140,141]. AS112 has been also shown to activate the complement system, a finding that permitted the establishment of a relationship between AS112 expression and PD, where complement activation had been observed before [142,143,144].

At the transcriptional level, we have shown that *SNCA112* is specifically overexpressed in cortical regions of patients with the pure form of DLB [145,146]. Unexpectedly, this isoform was downregulated in the frontal cortices of cDLB and AD patients, indicating that *SNCA112* plays a specific role in pDLB on the one hand, and that pDLB and cDLB may develop by different primary mechanisms, on the other [146].

A few years ago, we reported that *SNCA112* mRNA is specifically overexpressed in the frontal cortex of pDLB patients [145,146]. Moreover, this isoform is downregulated in the frontal cortices of cDLB and AD patients, but is slightly upregulated in PD patients [146]. These data were extended by a study of McLean and collaborators [147], who analyzed *SNCA* isoform expression in PD brain, where *SNCA112* was overexpressed in the substantia nigra and the cerebellum. When analyzed in human *SNCA* expressing mice, *SNCA112* expression was higher in brain of *SNCA* expressing mice than in controls, and expression of *SNCA112* increased with age, especially in the ventral midbrain [147].

*SNCA* splicing has been also studied in MSA brain, where all three alternative transcripts, exon 3-lacking, exon 5-lacking, and exon-3-and-5-lacking transcripts could be detected. *SNCA112* was drastically overexpressed in substantia nigra, striatum, cerebellar cortex, and nucleus dentatus of MSA cases when compared to controls, but also to PD [148]. This overexpression was also observed in the prefrontal cortex, where *SNCA140* levels were similar to controls and PD [148]. Recently, it has been shown that misfolded AS may occur as different strains, as ribbons or fibrils, and that both show different levels of neurotoxicity, seeding, and propagation [149]. Differences in AS antibody epitope recognition of LBs in PD, and GCIs in MSA [150,151,152], as well as in the characteristics of insoluble AS isolated from the PD and MSA brain, indicate that the brains of these patients may contain different strains [153,154]. These observations, together with the finding that *SNCA112* is drastically overexpressed in MSA brain, may suggest that *SNCA112* could be a specific component of the MSA-specific AS strain.

In a recent biomarker study, *SNCA112* expression has been analyzed in blood of 202 de novo PD cases and 138 healthy controls. In PD, *SNCA112* expression was decreased in 19% compared to controls, indicating for the first time that *SNCA112* could be a peripheral biomarker for a synucleinopathy [155].

Finally, it has been also shown that at least three single-nucleotide polymorphisms (SNPs), rs356219 at the *SNCA* 3’end, rs365165 in the 3’UTR, and rs2736990 in intron 5, alter *SNCA112/SNCA140* mRNA expression ratios in the frontal cortex [156]. The three SNPs are composed of A- and G-alleles, and *SNCA112/SNCA140* expression ratio increases in the presence of G-alleles, showing AA genotype carriers low, AG genotype carriers intermediate, and GG genotype carriers high expression ratios [156]. Accordingly, association analysis between rs356219 and PD revealed that the G-allele confers risk to develop PD, and the A-allele protects against it [156].

#### 2.2.2. In-Frame Splicing of Exon 3

AS126 occurs as a result of the in-frame splicing of *SNCA* exon 3, producing a 14 amino acid deletion that involves part of the linker between both N-terminal helices, as well as the N-terminal part of the central helix (Figure 1) [37]. As a result, alterations in both helices, include the shortening of the highly amyloidogenic NAC region, which is primarily involved in AS oligomerization and aggregation. Interestingly, four of the five PD-related AS mutations (E46K, H50Q, G51D, and A53T), and the MSA-related mutation, A53E, are located in exon 4, constituting the mutation hotspot of *SNCA*. 

Since the C-terminal structure of AS126 remains intact, its net charge is even lower than that of AS140 (−10.2 vs. −9). Therefore, it could be expected that low net charge of AS126 diminishes its aggregation properties, conferring anti-aggregation characteristics to this isoform [105]. Accordingly, in a HEK294T cell model, AS126 exhibited low aggregation rates when expressed in absence, or together with AS140 [136]. However, AS126 was able to form fibrils, in vitro, which were shorter than AS140 fibrils, but were arranged in parallel arrays [136]. As expected, AS126 showed a reduced ability to bind plasma membranes, due to the interruption of its protein membrane binding domain [137], and AS126 expression in yeast does not display toxicity in the absence of AS140. Instead, it seems to be able to induce AS140 toxicity as suggested by the results of AS140 and AS126 co-expression experiments, although to a lesser extent than AS112 [137]. 

There is some evidence suggesting that AS126 may play a protective role. First, we demonstrated that *SNCA126* mRNA levels are drastically diminished in the frontal cortices of DLB, but also AD brains. By contrast, the frontal cortex of PD brains showed increased *SNCA126* expression [145]. The DLB cases in our series had been neuropathologically classified as AS pathology stages 5 and 6, while the PD cases showed AS pathology stages 3 and 4 [26,157]. Whereas stages 5 and 6 correspond to the presence of Lewy pathology in the cerebral cortex, in stages 3 and 4, cortical regions remain unaffected. According to this staging, DLB brains present Lewy pathology, but diminished *SNCA126* levels, and PD brains do not show Lewy pathology, but elevated *SNCA126* levels in the frontal cortex. If AS126 is an aggregation-preventing isoform, its decrease in DLB could enhance the formation of Lewy pathology, and its increase in PD could prevent it [105].

In PD, *SNCA126* is also increased in the substantia nigra, but its expression does not differ in the cerebellum when compared to control brains [147]. Young human *SNCA* expressing mice show elevated *SNCA126* levels throughout the brain, being highest in the frontal cortex. During aging, these high *SNCA126* levels only remain in the ventral midbrain, where they even increase [147]. 

In MSA, *SNCA126* expression is drastically diminished in the substantia nigra and the striatum, and also decreased in the cerebellar cortex and the nucleus dentatus [148]. As mentioned in the previous section, *SNCA112* is importantly increased in these brain areas in MSA, creating a striking disequilibrium between the levels of both isoforms [148]. We had also observed a shift with *SNCA112* overexpression and *SNCA126* diminution in the frontal cortex of DLB, although to a lesser extent [145]. While minor *SNCA* isoforms constitute only about 5% of total *SNCA* [136], the combined expression change, comprising the increase of *SNCA112* accompanied by the decrease of *SNCA126*, could represent one of the factors participating in the seeding process of AS aggregation in synucleinopathies. 

After analyzing *SNCA126* expression in the brain and identifying disease-specific differences, we studied the DNA sequence surrounding *SNCA* exon 3 with the aim of identifying some regulatory element able to modify *SNCA126* expression. As a result, we detected a polyT sequence of variable length between positions −128 and −140 upstream to exon 3 [158]. This polyT sequence is composed of three alleles: the 7T-allele, which is the most common in the general population, a shorter allele containing 5 T, and a larger allele of 12 T. When correlating with allele length, we found that the longer the polyT stretch, the higher *SNCA126* expression levels in the normal brain [158]. We also showed that 12T alleles are accumulated in healthy aging, and that 5T alleles are absent in healthy individuals older than 80 years [158]. An opposite tendency was observed in AD, where low *SNCA126* expression 5T alleles were accumulated in the oldest age group (over 80 years), and high *SNCA126* expression 12T alleles were diminished [158].

#### 2.2.3. In-Frame Splicing of Exons 3 and 5

The small AS variant AS98 is derived from *SNCA* transcripts lacking exons 3 and 5, and is characterized by the drastic shortening of its N-terminal (as seen in AS112) and the interruption of the C-terminal helical domain (as seen in AS126) [126]. The net charge of AS98 is −0.2, slightly lower than the AS112 net charge, but significantly higher than full-length AS or AS126 net charges [105]. In AS98, the NAC domain remains intact, and is, together with the N-terminal helix, the most prominent protein part. These merely structural observations may suggest that AS98, similar to AS112, enhances aggregation, and that it may be involved in the AS seeding process. 

Analysis of mRNA expression in the frontal cortex revealed that *SNCA98* levels were significantly increased in DLB, PD, but also AD when compared to controls [126]. In another study, *SNCA98* expression was analyzed in PD brains, and its expression was significantly higher in the substantia nigra and cerebellum than in the frontal cortex [147]. 

In human *SNCA* expressing mice, *SNCA98* levels were highest in the cortexes of young mice, and they decreased during aging. The contrary effect was observed in the ventral midbrain where low levels observed in young mice raised with aging [147]. Furthermore, the analysis of AS98 aggregation properties revealed that AS98, similar to AS112 and AS126, did not enhance the formation of aggresomes or multiple aggregates, either alone or in presence of AS140. But interestingly, recombinant AS98 formed circular pore-like structures in vitro [136].

Finally, the overexpression of *SNCA98* in transfected pheochromocytoma cells did not lead to the formation of detectable AS aggregates, but to an increase in the production of reactive oxygen species and lipid peroxidation [159]. 

As shown along Section 2.2, the revision of the literature related to *SNCA* splicing reveals that the different AS isoforms play differential roles in the pathogenesis of synucleinopathies. One of the most important events seems to be the shift of the isoform expression ratios favoring the formation and accumulation of altered AS species. 

### 2.3. 3’ Untranslated Region Splicing

An elevated degree of mRNA transcript variability is due to the alternative use of tandem 3’UTRs and polyadenylation sites, resulting in the generation of isoforms that contain either short or long 3’UTR. Consensus sequences targeted by the signal transduction and activation of RNA splicing (STAR) factors are present in transcripts subjected to alternative polyadenylation [98]. Signal transduction and activation of STAR factors and neuron specific splicing factors, such as NOVA proteins, are involved in alternative inclusion of larger 3’UTR portions in mRNAs of known genes [160,161,162]. Consensus sequences and binding motifs for both are significantly enriched in 3’UTR regions [163]. In neurons, alternative polyadenylation generates mRNA isoforms with different subcellular localization and function. For example, transcripts of brain-derived neurotrophic factor (BDNF) with long 3’UTR are specifically targeted to dendrites regulating their morphology, whereas transcripts with short 3’UTR mostly remain within the cell soma [164]. It has been also shown that a change in the polyadenylation site is a common mechanism in response to neuronal activity in cortical neurons [165]. In this context, transcripts of the transcription factor myocyte enhancer factor-2 (MEF2) produced in stimulated and unstimulated neurons, differ only in their 3’UTR lengths [166]. 

A recent study has reported the existence of at least five *SNCA* transcripts differing in their 3’UTR length, that ranged between 290 and 2520 base pairs (bp) [106]. *SNCA* with 3’UTRs of 590 and 2520 bp are the most common, and all UTRs longer than 590 bp can be considered as long 3’UTR *SNCA* transcripts. In the normal brain, long 3’UTR *SNCA* expression correlates with a sub-network of gene transcripts that are associated with synaptic and vesicular transport. By contrast, in PD, a global rewiring is observed, and long 3’UTR transcripts correlate with nuclear localization and transcriptional regulation [106]. An increase of the ratio between long 3’UTR *SNCA* and the remaining *SNCA* transcripts has been found in PD, but not in the brain of controls, or patients with other neurodegenerative disorders. Moreover, the C-allele of rs356168, a SNP located about 3 kb downstream to the *SNCA* 3’UTR, is highly predictive for that increased ratio [106]. Finally, functional studies revealed that long 3’UTR *SNCA* are associated with AS localization and accumulation in mitochondria [106]. 

Expression levels of these transcripts were also analyzed in a recent multicenter study that explored their utility as blood biomarker for early PD. Whereas the overall diminution of *SNCA* transcripts was detected in blood of three independent early PD cohorts, long 3’UTR *SNCA* transcripts were especially diminished in disease [155]. 

Although the 3′UTR is not translated into protein, this sequence contains recognition sites for microRNAs (miRNAs), which are endogenous small noncoding RNAs that regulate gene expression post-transcriptionally [167]. miRNAs primarily bind to their target mRNA at the 3′UTR, promoting repression of mRNA translation [168,169]. Additionally, miRNAs also induce mRNA decay by recruiting deadenylases and decapping factors onto the target mRNAs through GW182/TNRC6 [170]. mRNA with longer 3’UTR are less stable because they carry more regulatory sites, resulting in higher mRNA degradation rates and lower translation levels [171]. 

**Figure 3:**
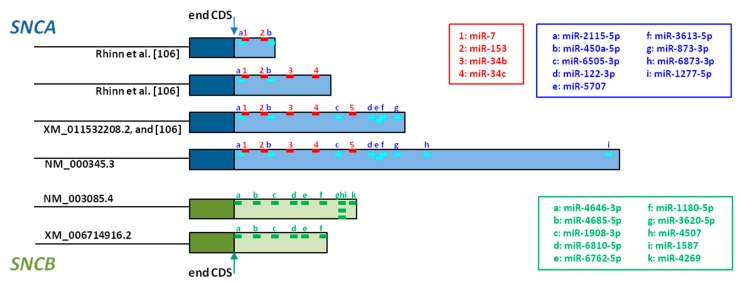
**Schematic representation of the 3’UTR of *SNCA* (blue) and *SNCB* (green).** *SNCA* and *SNCB* transcripts that differ in their 3’UTR lengths are shown. Of the four SNCA transcripts, two have been described in the literature, but have not yet been included in the NCBI database. Colored short bars represent the miRNA binding sites, red—experimentally confirmed, blue—predicted for *SNCA* [178], green—predicted for *SNCB*, at the same website. CDS: coding sequence.

Five miRNAs that directly regulate *SNCA* expression have been described so far [172]. miR-7 is the most studied, and its direct binding to *SNCA* 3’UTR has been confirmed in in vitro models, where transfection of HEK293T cells with miR-7 induced diminution of AS and inhibition of miR-7 led to increased AS expression [173,174]. Additional studies addressed the neuroprotective effect of miR-7, showing protection against H_2_O_2_-induced cell death in A53T-AS cells [173], and against MPP^+^-treatment in SH-SY5Y cells through activating the mTOR pathway [175]. miR-153 also binds directly to *SNCA* 3’UTR (Figure 3), has a direct effect on *SNCA* expression [174], and its overexpression in primary cortical neurons attenuated MPP^+^-induced neurotoxicity [175]. miR-34b and miR-34c also target *SNCA* (Figure 3). Their overexpression in SH-SY5Y cells reduced AS levels, and on the contrary their inhibition, increased AS levels, and the formation of AS positive aggregates in dopaminergic neurons [176]. Although the binding site for the fifth miRNA, miR-214, has not been identified so far, *SNCA* regulation by this miRNA has been shown in SH-SY5Y cells [177].

Different miRNA target prediction tools permit the analysis of mRNA 3’UTR regions. Whereas the database miRDB predicts 97 miRNAs that target *SNCA* [179], 9 different miRNA target *SNCA* when searching with miRBase [178]. The putative binding sites of these 9 miRNAs, together with the confirmed binding sites of the miRNAs discussed above, are represented in Figure 3, and whereas *SNCA* transcripts including the shortest 3’UTR contain only 4 miRNA binding sites, *SNCA* transcripts with the longest 3’UTR contain 13 miRNA binding sites. This observation confirms that transcripts with longer 3’UTR are targeted by many more miRNAs than transcripts with short 3’UTR [170,171]. 

## 3. *SNCB* Alternative Splicing and Its Role in Synucleinopathies

Although Rockenstein and colleagues [180] described decreased *SNCB* mRNA levels in AD and DLB when compared to controls, so far, the expression of the different *SNCB* transcript variants has been studied to a much lesser extent than those of *SNCA*. 

### 3.1. 5’ Untranslated Region Splicing

Alternative inclusion of one or two initial exon in this untranslated region is reported for *SNCB* (Figure 1 and Figure 2) [110]. The specific inclusion/lack of exon 2 gives rise to the two main transcripts described: *SNCBtv1* containing untranslated exon 2 in addition to exon 1 (NCBI accession: NM_001001502), and *SNCBtv2* lacking exon 2 (NCBI accession: NM_003085). Affecting the 5’-UTR, both transcripts are translated into the same protein without altering its function and its disease-involvement [181]. Both *SNCB* transcript variants are expressed in the brain, but not in blood, and our previous studies have shown that *SNCBtv2* expresses 50 times more than *SNCBtv1* in the frontal cortex and 10 times more in both the temporal cortex and the caudate nucleus of control brains [182]. 

When analyzed in three areas of DLB, PDD, AD and control brains, results revealed that both *SNCB* transcripts are not expressed in the temporal cortex of pDLB brain, although an important diminution of *SNCBtv2* was also observed in cDLB, PDD, and AD [182]. In the temporal cortex of PD without dementia, only a very slight diminution of *SNCB* transcripts was observed [183]. In the frontal cortex, pDLB brains also showed lowest expression levels of both *SNCB* transcripts, in cDLB, diminution, but mainly of *SNCBtv1*, was found. Interestingly, the caudate nucleus presented a different expression profile with overexpression of *SNCBtv1*, mainly in cDLB and both PD forms [181,182]. These results permitted us to postulate that pDLB constitutes a molecular subgroup of DLB, and is characterized by the drastic diminution of BS levels in the cortex, an aggressive disease course, and pure Lewy pathology in the brain [182]. Thus, considering BS as “natural inhibitor” of AS oligomerization and aggregation deposition [8,82], the lack of BS in the cortex of these brains could be the primary cause for the development of the disease in these cases [182]. 

With the aim to identify the possible cause of this diminution in *SNCB* expression, we also analyzed the promoter region of the *SNCB* gene. It contains, similar to the *SNCA* promoter, a CpG island with a length of 900 bp [182]. Differently to the *SNCA* CpG island located in intron 1, the *SNCB* CpG island spans from 200 bp upstream to exon 1, includes exon 1 and intron 1, to exon 2 (Figure 2). Although we were not able to identify significant methylation levels in the temporal cortex, further studies addressing in-depth analysis of this region are needed to rule out the involvement of *SNCB* promoter methylation in the development of pure DLB. 

### 3.2. Exon Skipping

Considering the homology between *SNCA* and *SNCB* genes, we have also examined if *SNCB* splicing bears *SNCA*-like transcripts lacking exons 3 or 5 [183]. As a result, we identified exon 3 lacking transcripts that would give rise to a 120 amino acid protein, BS120, and exon 5 lacking transcripts, producing a 104 amino acid protein *SNCB104*. These splice variants represent minor transcripts and constitute about 1% of total *SNCB* in the temporal cortex and caudate nucleus, and about only 0.5% in the frontal cortex. Whereas *SNCB120* is produced from two transcripts, containing or not 5’UTR exon 2, *SNCB104* only occurs as a transcript that also lacks exon 2 [184]. Moreover, *SNCB104* is absent in the caudate nucleus, while *SNCB120* was expressed in 10 different brain areas of control brains. 

When studied in disease, important dysregulation of alternative splicing was detected in the cortex of pDLB brains, with diminution of both *SNCB120* and *SNCB104* in the frontal cortex and of *SNCB120* in the temporal cortex. In the other disease groups, including cDLB, PDD, and AD, no significant changes were detected [184]. Similar to the results found for 5’UTR varying transcripts, the caudate nucleus presented its own expression profile with overexpression of *SNCB120* in cDLB and PDD. Moreover, *SNCB104*, whose expression could not be detected in the caudate nucleus of control brains, was detected in the caudate nucleus of both DLB forms [184].

These data indicated that lack of *SNCB* transcripts in cortical areas is a possible disease promoting characteristic in DLB, and that the caudate nucleus shows a very own *SNCB* expression profile. 

### 3.3. 3’ Untranslated Region Splicing

So far, there are no studies addressing the possible variability of the 3’UTR of the *SNCB* gene. Although NCBI reports *SNCB* transcripts with three different 3’UTRs [110], these have not been investigated so far, so that future studies should undertake this task to clarify their role in disease development. 

As mentioned before, the *SNCB* gene has been much less studied than *SNCA*. Although no miRNAs have been reported for *SNCB* regulation, 10 miRNAs could interact potentially with *SNCB* transcripts with large 3’UTR (Figure 3) [178]. In contrast, *SNCB* transcripts with short 3’UTR would be regulated by 6 of these miRNAs (Figure 3). This fact underlines, once more, that mRNA transcripts with longer 5’UTR are more likely to undergo post-transcriptional regulation by miRNAs and mRNA decay, than transcripts with short 3’UTR [170,171]. 

## 4. Conclusions

Both *SNCA* and *SNCB* genes belong to the same gene family, and are characterized by an elevated homology in their amino acid content but also in their gene structure. As a result of this homology, they undergo similar splicing events that affect all three gene regions, 5’UTR, coding sequence and 3’UTR, and the result is an elevated diversity of transcripts produced from both *SNCA* and *SNCB*. 

α-Synuclein aggregation is known as the major pathological event leading to the development of synucleinopathies, including PD, DLB and MSA. Different studies carried out over the past 10 years have consistently shown that the dysregulation of *SNCA* and *SNCB* alternative splicing constitutes a trigger, especially for the development of pDLB and MSA, but is also involved in the development of the other disease groups.
